# Optimizing Thermomechanical Processing for Producing Bulk Fine-Grained Aluminum Alloy with Thermal Stability

**DOI:** 10.3390/ma18174180

**Published:** 2025-09-05

**Authors:** Jesada Punyafu, Chonlada Domrong, Ussadawut Patakham, Mitsuhiro Murayama, Chaiyasit Banjongprasert

**Affiliations:** 1 Department of Physics and Materials Science, Chiang Mai University, Chiang Mai 50200, Thailand; punyafu.jesada.397@m.kyushu-u.ac.jp (J.P.); chonlada.dom1990@gmail.com (C.D.); 2 Institute for Materials Chemistry and Engineering, Kyushu University, Fukuoka 816-8580, Japan; murayama@vt.edu; 3 National Metal and Materials Technology Center, National Science and Technology Development Agency, Pathum Thani 12120, Thailand; ussadawp@mtec.or.th; 4 Department of Materials Science and Engineering, Virginia Tech, Blacksburg, VA 24061, USA; 5 Center of Excellence in Materials Science and Technology, Chiang Mai University, Chiang Mai 50200, Thailand

**Keywords:** aluminum alloy, fine-grained structure, severe plastic deformation (SPD), equal channel angular pressing (ECAP), electron microscopy, thermal stability

## Abstract

This study investigates the thermal stability of fine-grained structures achieved through different severe plastic deformation (SPD) and heat treatment paths. Bulk fine-grained Al-0.1Sc-0.1Zr (wt%) alloy was produced via equal channel angular pressing (ECAP) using routes Bc or C, with aging before or after the ECAP. Electron back-scattered diffraction (EBSD) and transmission electron microscopy (TEM) analyses demonstrate excellent thermal stability of all four specimens. They maintain mean grain sizes below 5 μm after a 10 h thermal test at 450 °C, attributed to the presence of nano Al_3_(Sc,Zr) precipitates within the microstructures. Route Bc in the ECAP method forms more stable high-angle grain boundaries (HAGBs) than route C. Whether aging occurs before or after the ECAP, similar microstructural changes are observed after thermal testing, allowing fine-tuning of the microstructure depending on the application or subsequent processes.

## 1. Introduction

Severe plastic deformation (SPD) efficiently produces bulk fine-grained metallic materials with superior mechanical properties compared to coarse-grained counterparts [1]. However, high densities of lattice defects in SPD-processed microstructures limit ductility [2]. While subsequent heat treatments can improve ductility, they often decrease yield strength due to grain coarsening [3]. Thus, small amounts of scandium (Sc) and zirconium (Zr) are added to aluminum (Al), and aging is performed to promote Al_3_(Sc,Zr) nano precipitates, which efficiently impede grain boundary movement and help maintain the fine-grained structures (mean grain sizes < 10 μm) at elevated temperatures [4]. However, the impact of aging before or after the SPD process on thermal stability remains uncertain due to its potential effect on precipitate formation.

Equal channel angular pressing (ECAP) is a widely considered SPD technique for producing bulk fine-grained metals for industrial-scale manufacturing [5,6]. By repetitively pressing bulk materials through a die containing two channels, a highly uniform fine-grained microstructure is achieved. Recent studies have revealed that this microstructure develops through a complex interplay of dislocation accumulation, the formation of low-angle and high-angle grain boundaries, and dynamic recrystallization [7,8]. This fine-grained microstructure offers several advantages; for instance, recent studies have demonstrated the potential use of fine-grained aluminum alloys as anodes in aluminum-air batteries [9,10]. Among ECAP parameters, the rotation of specimens between passes—routes A, Bc, and C rotating 0°, 90°, and 180°, respectively—significantly influences microstructure evolution [11]. Although it is still debated whether route Bc or C is the most effective route for grain refinement, route A is the least effective one [12,13,14,15]. Moreover, Cabibbo [15] noted that the microstructure of pure Al produced via route C demonstrates greater stability than route Bc under annealing. However, whether this holds for Al alloys containing Al_3_(Sc,Zr) precipitates remains unclear.

This study aims to find the best ECAP route and aging sequence to maximize the microstructure thermal stability of fine-grained Al alloys containing Al_3_(Sc,Zr) precipitates. It focuses on producing bulk fine-grained Al-0.1Sc-0.1Zr (wt%) specimens through ECAP using routes Bc or C. Aging was conducted before and after the ECAP; subsequently, the thermal stability of these specimens was evaluated. Microstructures and precipitates were analyzed using electron microscopy techniques.

## 2. Materials and Methods

**Figure 1:**
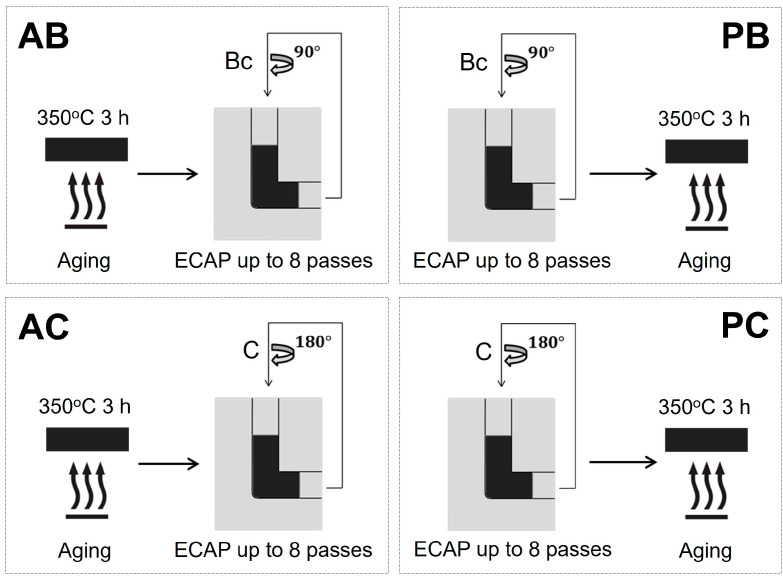
Schematic of different thermomechanical processes for producing AB, PB, AC, and PC specimens. ECAP = equal channel angular pressing.

An Al-0.1Sc-0.1Zr alloy (wt%) was prepared for this study. After casting, the alloy was solution heat treated at 620 °C for 48 h, machined into rod-shaped bars, 20 mm in diameter and 100 mm in length, and subjected to different thermomechanical processes, as illustrated in Figure 1. The different thermomechanical routes are abbreviated as follows: AB = aging followed by ECAP via route Bc, PB = ECAP via route Bc followed by aging, AC = aging followed by ECAP via route C, and PC = ECAP via route C followed by aging. Aging was performed at 350 °C for 3 h. This aging condition was selected because it has been reported to promote a uniform distribution of coherent Al_3_Sc precipitates within the fine-grained microstructure of Al-Sc alloys [16,17,18,19]. ECAP was performed at room temperature using an ECAP die (Φ = 90°, Ψ = 20°) up to eight passes (equivalent strain = 8.4). The bars were rotated 90° and 180° clockwise between passes for routes Bc and C, respectively. All specimens underwent a thermal test at 450 °C for 10 h to assess their thermal stability. This condition was chosen to evaluate long-term stability beyond typical short-term tests (about 1 h) at 300–450 °C [4,16,19,20].

Microstructure characterization was conducted using electron back-scattered diffraction (EBSD) and transmission electron microscopy (TEM) on the cross-sectional area of the specimens. EBSD samples were prepared via electropolishing and analyzed using FEI Nova NanoSEM 450 (Thermo Fisher Scientific, Hillsboro, OR, USA), Hitachi SU8230 (Hitachi High-Tech Corporation, Tokyo, Japan), and JEOL JSM-IT300 (JEOL Ltd., Akishima, Tokyo, Japan), operating at 20 kV with step sizes of 50–150 and 300–350 nm before and after thermal testing, respectively. TEM samples were prepared by electropolishing or cryo-ion slicing and analyzed using JEOL JEM-2010 and ARM-200F (JEOL Ltd., Akishima, Tokyo, Japan) operated at 200 kV.

## 3. Results and Discussion

**Figure 2:**
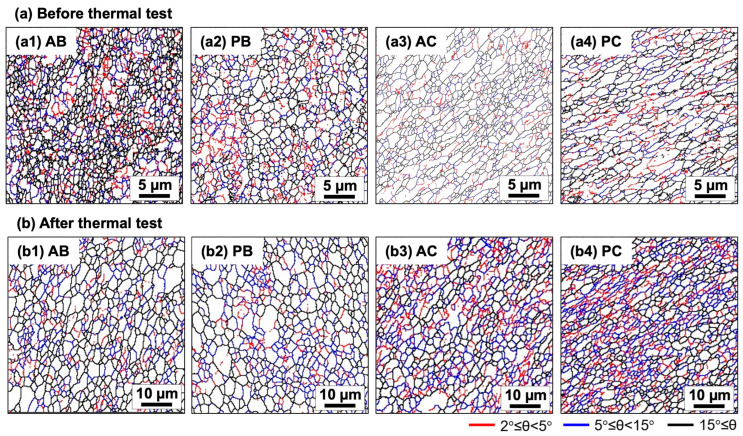
Boundary misorientation maps of specimens (**a**) before and (**b**) after the thermal test at 450 °C for 10 h. Low, medium, and high-angle grain boundaries (HAGBs) with misorientations (θ) of 2° ≤ θ < 5°, 5° ≤ θ < 15°, and 15° ≤ θ are represented by red, blue, and black lines, respectively.

**Figure 3:**
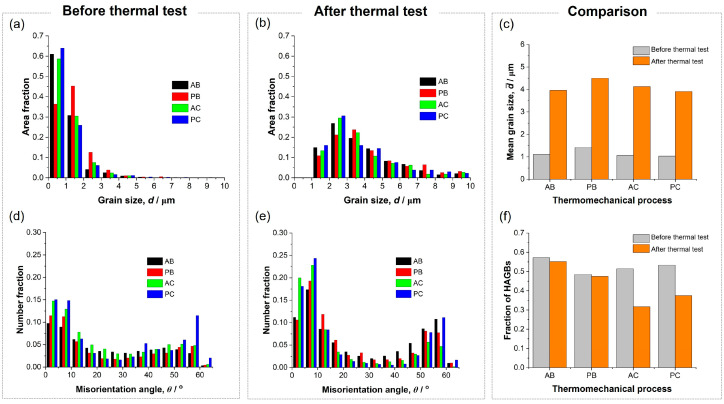
(**a**,**b**) Distribution of grain sizes, and (**c**) comparison of mean grain sizes of specimens before and after the thermal test. (**d**,**e**) Distribution of misorientation angles, and (**f**) comparison of fraction of HAGBs of specimens before and after the thermal test.

Figure 2a depicts the microstructures of specimens before the thermal test. Specimens produced via route Bc (AB and PB specimens) exhibited equiaxed grains, whereas those produced via route C (AC and PC specimens) showed elongated grains. This distinction was attributed to variations in shearing characteristics and fine-grained formation between ECAP via routes Bc and C [21]. Nevertheless, all specimens demonstrated a comparable grain size distribution (Figure 3a) with approximately 1 µm mean values (Figure 3c) and a similar distribution of misorientation angles (Figure 3d), with about half characterized as high-angle grain boundaries (HAGBs) (Figure 3f). Thus, besides grain morphology, ECAP utilizing routes Bc and C produced similar characteristics of fine-grained structures.

**Figure 4:**
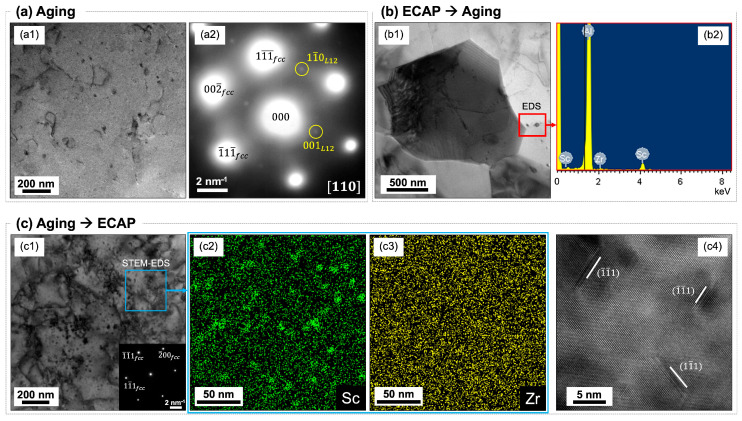
(**a1**) BF-TEM image and (**a2**) corresponding SAED pattern of precipitates promoted before ECAP. (**b1**) BF-TEM image and (**b2**) corresponding EDS spectrum of precipitates promoted after ECAP. (**c1**) BF-TEM image with corresponding SAED pattern, STEM-EDS elemental maps for (**c2**) Sc and (**c3**) Zr, and (**c4**) HRTEM image showing dislocation loops obtained from a specimen subjected to aging and then ECAP.

After the thermal test, the grains in all specimens became equiaxed (Figure 2b). The grain size distribution shifted toward larger values in all specimens (Figure 3b), with mean values increasing to approximately 4 μm (Figure 3c). Notably, compared to fine-grained pure Al and Al–Mg alloys processed by ECAP, the investigated alloy exhibited enhanced thermal stability (resistance to grain coarsening upon heating). For instance, grain coarsening to >10 μm typically occurs in those alloys after heating at 300 °C for 1 h [20]. This improved thermal stability—defined here as the suppression of grain growth around the recrystallization temperature of pure Al (0.3 to 0.4 of its melting point)—was attributed to the presence of thermally stable Al_3_(Sc,Zr) nano-precipitates within the microstructure, which effectively impedes grain boundary migration and thereby slow down recrystallization and grain coarsening [4]. Figure 4 shows various forms of Al_3_(Sc,Zr) phase under different thermomechanical conditions. Figure 4(a1) depicts a bright-field (BF) TEM image showing a few 10 nm-sized spherical precipitates formed in a specimen solely aged at 350 °C for 3 h. The corresponding selected area electron diffraction (SAED) pattern (Figure 4(a2)), taken near the [110] zone axis, reveals extra diffraction spots in addition to the fundamental aluminum reflections. These extra spots correspond to the L1_2_-ordered structure of the Al_3_(Sc,Zr) phase. Figure 4(b1) illustrates a trend of precipitate growth after ECAP processing via route Bc and then aged (ECAP → aging). The energy-dispersive X-ray spectroscopy (EDS) spectrum (Figure 4(b2)) indicates characteristic X-ray peaks corresponding to Sc and Zr. This demonstrates that the 350 °C aging for 3 h after SPD leads to precipitate growth [22]. This growth occurs due to the high density of lattice defects—such as dislocations and grain boundaries—introduced by ECAP, which accelerates diffusion and promotes precipitate growth during subsequent aging. Figure 4(c1) depicts a BF-TEM image showing a representative general microstructure of the specimen that was aged and then processed by ECAP via route Bc (aging → ECAP). While some fine dark spherical contrasts were observed, no diffraction spots corresponding to the L1_2_ structure were found in the corresponding SAED pattern. However, the scanning TEM (STEM)-EDS elemental mapping from the area indicated in Figure 4(c1) reveals the clustering of Sc atoms (Figure 4(c2)) and a uniform distribution of Zr atoms (Figure 4(c3)). This indicates that SPD after aging promotes a partial dissolution of Al_3_(Sc,Zr) precipitates [23,24,25]. This dissolution occurs due to the high defect density introduced by ECAP, which enhances atomic mobility even at room temperature, facilitating the redistribution of solute atoms from the precipitates into the matrix. Since the dark spotty contrasts in the BF-TEM image do not overlap with the Sc clusters observed in the EDS map, these appear to be strain contrasts around dislocation loops, as confirmed by the high-resolution TEM (HRTEM) image (Figure 4(c4)).

Figure 3e illustrates the changes in the distribution of misorientation angles after thermal testing. The fraction of misorientation angles between 2° and 15° increased in all specimens, indicating grain coalescence, a phenomenon contributing to grain coarsening in metals processed via SPD during subsequent heat treatments [26]. While the fraction of HAGBs in the route Bc-processed specimen decreased modestly after the thermal test, the route C-processed specimen showed a more noticeable change (Figure 3f). These results indicate that HAGBs generated via route Bc exhibit greater stability under high temperatures than those produced via route C. This discrepancy may have stemmed from variations in microstructural evolution during repetitive ECAP between the two routes. Specifically, although the microstructure is developed through two distinct and intersecting sets of shear planes in route Bc, it is formed via a single set of shear planes in route C [11,14,15,21]. Consequently, thermally stable Sc and Zr solutes, along with Al_3_(Sc,Zr) precipitates, are expected to be more uniformly distributed in the microstructure of the route Bc-processed specimen. This uniform distribution likely contributed to more thermally stable HAGBs and a smaller reduction in HAGB fraction after the thermal test in route Bc specimens. Therefore, ECAP using route Bc is recommended for producing fine-grained Al alloys with thermal stability.

Aging before or after ECAP resulted in similar thermal stability. As depicted in Figure 3, specimens aged before or after ECAP exhibited similar trends in grain size distributions, mean grain sizes, misorientation angle distributions, and fractions of HAGBs following the thermal test. Although aging before ECAP resulted in partially dissolved precipitates (Figure 4c) while aging after ECAP resulted in coarsened precipitates (Figure 4b), both forms contributed to similar microstructural changes during the thermal test. Therefore, this study indicates that aging to promote nano Al_3_(Sc,Zr) precipitates within the microstructure can be conducted before or after ECAP without compromising the microstructure thermal stability of the alloy’s microstructure.

## 4. Conclusions

We produced bulk fine-grained Al-0.1Sc-0.1Zr (wt%) specimens via ECAP using routes Bc or C, with aging before or after the ECAP, then tested their thermal stability at 450 °C for 10 h. Mean grain sizes of all specimens were <5 μm after the thermal test due to nano Al_3_(Sc,Zr) precipitates. HAGB fractions notably decreased in specimens produced via route C compared to route Bc. Aging before or after ECAP yielded similar microstructural changes. These findings advocate for utilizing ECAP via route Bc to produce bulk fine-grained Al alloy with enhanced thermal stability while aging can be conducted either before or after the ECAP.

## Data Availability

The original contributions presented in this study are included in the article. Further inquiries can be directed to the corresponding author.
